# Risk Factors for Death in Children with Visceral Leishmaniasis

**DOI:** 10.1371/journal.pntd.0000877

**Published:** 2010-11-02

**Authors:** Márcia Jaqueline Alves de Queiroz Sampaio, Nara Vasconcelos Cavalcanti, João Guilherme Bezerra Alves, Mário Jorge Costa Fernandes Filho, Jailson B. Correia

**Affiliations:** 1 Instituto de Medicina Integral Prof. Fernando Figueira (IMIP), Recife, Brazil; 2 Faculdade Pernambucana de Saúde, Recife, Brazil; 3 Faculdade de Ciências Médicas, Universidade de Pernambuco, Recife, Brazil; 4 Universidade Federal de Pernambuco, Recife, Brazil; Emory University, United States of America

## Abstract

**Background:**

Despite the major public health importance of visceral leishmaniasis (VL) in Latin America, well-designed studies to inform diagnosis, treatment and control interventions are scarce. Few observational studies address prognostic assessment in patients with VL. This study aimed to identify risk factors for death in children aged less than 15 years admitted for VL treatment in a referral center in northeast Brazil.

**Methodology/Principal Findings:**

In a retrospective cohort, we reviewed 546 records of patients younger than 15 years admitted with the diagnosis of VL at the Instituto de Medicina Integral Professor Fernando Figueira between May 1996 and June 2006. Age ranged from 4 months to 13.7 years, and 275 (50%) were male. There were 57 deaths, with a case-fatality rate of 10%. In multivariate logistic regression, the independent predictors of risk of dying from VL were (adjusted OR, 95% CI): mucosal bleeding (4.1, 1.3–13.4), jaundice (4.4, 1.7–11.2), dyspnea (2.8, 1.2–6.1), suspected or confirmed bacterial infections (2.7, 1.2–6.1), neutrophil count <500/mm^3^ (3.1, 1.4–6.9) and platelet count <50,000/mm^3^ (11.7, 5.4–25.1). A prognostic score was proposed and had satisfactory sensitivity (88.7%) and specificity (78.5%).

**Conclusions/Significance:**

Prognostic and severity markers can be useful to inform clinical decisions such as whether a child with VL can be safely treated in the local healthcare facility or would potentially benefit from transfer to referral centers where advanced life support facilities are available. High risk patients may benefit from interventions such as early use of extended-spectrum antibiotics or transfusion of blood products. These baseline risk-based supportive interventions should be assessed in clinical trials.

## Author Summary

Visceral leishmaniasis (VL) is a deadly disease caused by a protozoan called *Leishmania*. It is transmitted to humans from infected animals by a sandfly bite. Most people actually manage to control the infection and do not get sick, while others develop a range of symptoms. VL impairs the production of blood components and causes the immune system to malfunction, thus anemia, bleeding, and bacterial infections often complicate the disease and can lead to death. To identify risk factors for death from VL, the authors studied 546 children in a referral center in Recife, Brazil. They looked at clinical history, physical examination and full blood counts on the assumption these could be easily assessed in peripheral health facilities. They found that the presence of fast breathing, jaundice, mucosal (e.g. gum) bleeding and bacterial infections would each increase the risk of death in three to four-fold. The presence of very low counts of neutrophils and platelets would increase the risk of death in three and 12-fold respectively. This knowledge can help clinicians to anticipate the use of antibiotics or transfusion of blood products in high risk patients, who would potentially benefit from transfer to centers with advanced life support facilities.

## Introduction

In Latin America, visceral leishmaniasis (VL) cases occur from Mexico to Argentina, but around 90% of reported cases come from Brazil [1]. Despite substantial underreporting in several different parts of the world [1], Brazil's national surveillance system has informed estimates of disease burden in this country, where VL cases are found in the Distrito Federal and in 22 of 26 states [2]. Between 1990 and 2008 nearly 58,000 cases were reported in Brazil, but annual numbers have increased from 1,944 cases in 1990 to 3,990 in 2008 [3]. While the average VL incidence has been around 2 cases per 100,000 inhabitants [2], these figures do not take into account the focal nature of disease, which selectively pose a much higher burden on neglected populations from rural Northeast or from the slums around large cities, where major epidemics have recently occurred [4]. Although many infected individuals present no signs of disease, symptomatic VL can be very severe and its mortality remains substantial. Studies held in different parts of Brazil have reported case-fatality rates ranging from 4.4% to 10.2% in treated patients [5]–[7].

Despite the major public health importance of VL in Latin America, a recently published systematic review has shown that well-designed studies to inform diagnosis, treatment and control interventions in the region are scarce [8]. Unfortunately, national guidelines and treatment recommendations are often based on expert opinion instead of hard data from controlled trials [9]. Moreover, few observational studies address risk assessment in patients with the disease. For instance, as far as prognostic indicators are concerned, published evidence tend to come from elsewhere other than Latin America [10]–[13], from where we found only one report [5]. This report from Teresina (Brazil) identifies young age as a risk factor for poor prognosis, but it included patients from all ages in the analysis. In pediatric practice, however, it would be useful to identify children under higher risk of dying so that the best supportive and therapeutic options could be offered. The aim of the present study is to identify risk factors for death in children under the age of 15 years admitted for VL treatment in a referral center in northeast Brazil.

## Methods

### Ethics statement

The protocol was approved by the Research Ethics Committee of the Instituto de Medicina Integral Prof. Fernando Figueira (IMIP). Because data were retrieved from hospital records dating up to more than ten years and of patients whose families live away from Recife, the Ethics Committee agreed it would not be feasible to obtain individual signed consent forms. Data were however analyzed anonymously.

### Objectives

Socio-demographic, clinical and laboratory variables were assessed for association with death (primary outcome) in children and adolescents admitted for VL treatment. In addition, the study aimed to describe the case-fatality rate and the main causes of death in this group.

### Study design and participants

A retrospective cohort of children and adolescents with up to 14 years of age (inclusive) admitted for VL treatment was carried out at IMIP, a tertiary non-for-profit teaching hospital located in Recife (population 1.5 million), in the northeast of Brazil. IMIP, a referral center for the treatment of VL, belongs to the national public health system and provides free medical care for people from Pernambuco and neighbor states.

Patients notified to the hospital epidemiology department as having a diagnosis of VL were considered eligible to participate in the study and their records were reviewed, regardless of the outcome, from admission to discharge or death. Clinical, socio-demographic and laboratory variables were recorded onto a standardized form following a pilot study. Variables with more than 80% missing information were not included. Records were assessed by one of three medically-trained investigators and forms were reviewed for missing data and consistency. Data were entered twice into a computer database and EpiInfo *validate* module was used to identify inconsistencies. VL diagnosis was established either by confirmatory tests or by clinical and epidemiological criteria. The laboratory tests could be any one of the following: a positive Giemsa-stained bone marrow smear, a titer ≥1∶1,600 in direct agglutination test (DAT) or a titer ≥1∶40 in indirect immunofluorescence test (IFI). Diagnosis based on clinical and epidemiological criteria consisted of: a) clinical features of VL (fever and hepatosplenomegaly) *and* b) pancytopenia; *and* c) a positive epidemiological history *and* d) clinical response following a course of antimonials.

The weight/age Z scores (WAZ) were calculated using Nutrition Program, EpiInfo (Centers for Disease Control and Prevention/World Health Organization, 1978 reference). Children with WAZ <−3 were considered severely malnourished. Hemorrhagic manifestations were divided into 2 groups: cutaneous (bruising and petechiae) and mucosal (epistaxis and gengivorrhagia). Suspected bacterial co-infection was considered when the attending physician prescribed antibiotics and raised one of the following diagnoses: pneumonia, gastroenteritis, sepsis, otitis media and skin abscess, according to locally published guidelines [14]. Laboratory variables were categorized according to the following cutoff points: a) severe leucopenia (≤2,500/mm^3^); severe neutropenia (neutrophil count ≤500/mm^3^); b) severe anemia (hemoglobin <5 g/dL); and c) severe thrombocytopenia (platelet count <50,000/mm^3^).

### Sample size

A sample size calculation was performed in EpiInfo software. Assumptions were inferred from preliminary analysis of the first 150 patients and included an unexposed : exposed ratio of 2∶1 and a death rate of 5% in non-exposed. A total of 429 children would be required to detect a 2.5 increase in risk, with significance level of 5% and power of 80%.

### Statistical methods

Statistical analysis was performed using Epi-Info and STATA software packages. First, Chi-square tests were used to test the association between death and clinical, socio-demographic and laboratory variables in order to select those significant at a p level of <0.2 for inclusion in the logistic regression model in a stepwise backwards procedure. Variables showing an independent association with the risk of dying at the p<0.05 level remained in the final model.

The factors above listed as independent predictors of death from VL were used to build a prognostic score. All regression coefficients were divided by the lowest one (suspected bacterial co-infection) and rounded to the next integer without decimal points in order to facilitate clinical use. Subsequently, we validated the new scoring system with all the patients included in this study: the real outcome of each participant was compared to his/her prediction based on the new scoring system. Sensitivity, specificity, positive and negative predictive values and the area below the receiver operating characteristic curve (ROC) were calculated and used to evaluate the predictive performance of the prognostic score [15]. ROC reflects the ability of a scoring system to differentiate positive events (in this case, death in VL patients) and negative events (no death in VL patients). A higher ROC represents a better scoring system.

## Results

Between May 1996 and June 2006 a total of 557 patients were notified to the hospital epidemiology department as having a diagnosis of VL. Four records were not found and other 7 were excluded because, despite being initially notified as cases of VL, later had an alternative diagnosis (such as leukemia). Therefore, 546 patients are reported here. Out of these, 385 had diagnosis confirmed by a positive bone marrow smear, 14 by a positive DAT test, four by a positive IFI test and in 143 cases, diagnosis relied on clinical and epidemiological criteria.

Out of 546 patients, 275 (50%) patients were male. The median age was 3.2 years and the youngest child was four months-old and the eldest 13.7 years-old on admission. The median duration of symptoms was 30 days (interquartile range, 15–60 days). There were 57 deaths, with a case-fatality rate of 10%. The main immediate causes of death were associated bacterial infections in 21 (37%) cases, mucosal bleeding in 17 (29%), both infection and bleeding in 10 (18%) and infection or bleeding associated with liver failure in 8 (13%) and other causes in 2 (3%).

On univariate analysis (table 1), male gender, age less than five years-old, severe malnutrition, presence of diarrhea, edema, bleeding (cutaneous and mucosal), jaundice, dyspnea, suspected bacterial co-infection, hemoglobin <5 g/dL, leukocyte count <2,500/mm^3^, neutrophil count <500/mm^3^ and platelet count <50,000/mm^3^ were associated with risk of death with a p value <0.2 and were selected for inclusion in the multivariate logistic regression analysis.

**Table 1:** Factors associated with death from visceral leishmaniasis in children (univariate and multivariate analysis).

Variable	Dead(57)n(%)	Alive(489)n(%)	OR(95% CI)	Adjusted OR (95% CI)
**Clinical/demographic**				
Male sex	20(35)	255(52)	0.53 (0.3–1.0)	
Age <5 years old	47(82)	323(66)	3.19 (1.4–7.5)	
Severely malnourished*	8(14)	41(8)	1.78 (0.7–4.2)	
Diarrhea	11(19)	62(13)	1.73 (0.8–3.7)	
Edema	27(47)	69(14)	5.87 (3.1–11.1)	
Jaundice	16(28)	22(5)	8.71 (3.9–19.1)	4.4 (1.7–11.2)
Dyspnea	24(42)	61(13)	5.43 (2.8–10.3)	2.8 (1.2–6.1)
Mucosal bleeding	10(17)	10(2)	10.69 (3.7–28.2)	4.1 (1.3–13.4)
Cutaneous bleeding	18(32)	31(6)	6.82 (3.3–14.0)	
Upper gastrointestinal bleeding	4(7)	1(0.2)	38.27 (-)	
Associated infections	23(40)	56(11)	5.56 (2.9–10.7)	2.7 (1.2–6.1)
**Laboratory** †				
Total leukocyte count<2,500/mm^3^	21(37)	142(29)	1.41 (0.8–2.6)	
Total platelet count<50,000/mm^3^	43(75)	90(18)	15.01 (7.3–31.4)	11.7 (5.4–25.1)
Neutrophil count<500/mm^3^	17(30)	66(14)	2.68 (1.4–5.2)	3.1 (1.4–6.9)
Hemoglobin <5 g/dL	31(54)	103(21)	4.43 (2.4–8.1)	

*Weight not available for 2 children.

**†:** Full blood count missing for 3children.

Results of multivariate logistic regression analysis are shown on table 1. The presence of mucosal bleeding, jaundice, dyspnea, suspected bacterial co-infection, severe neutropenia (neutrophil count <500/mm^3^) and severe thrombocytopenia (platelet count <50,000/mm^3^) were identified as independent predictors of death from VL. Severe thrombocytopenia showed the strongest association with death (adjusted OR 11.7). The interactions between “mucosal bleeding” and “thrombocytopenia” as well as “neutropenia” and “suspected bacterial co-infection” were tested, but no statistically significant interactions were found (p = 0.21 and p = 0.11 respectively).

The generated scoring system (table 2) consisted in allocating the following points for each variable when present: one for dyspnea, suspected bacterial co-infection and severe neutropenia; two for mucosal bleeding and jaundice; three for severe thrombocytopenia. Any patient could have a score ranging from 0 to 9. A score ≥3 was selected as the best predictor of death because it was able to gather the most adequate combination of sensitivity (88.7%), specificity (78.5%), positive predictive value (32.0%), negative predictive value (78.5%) and area under ROC curve (89.5%).

**Table 2:** Prognostic scoring system.

Variable	Score
Dyspnea	1
Associated infections	1
Neutrophil count <500/mm^3^	1
Jaundice	2
Mucosal bleeding	2
Total platelet count <50,000/mm^3^	3

Allocate the points above for each variable when present.

## Discussion

The present study describes four clinical (mucosal bleeding, jaundice, dyspnea, suspected bacterial co-infection) and two laboratory variables (severe neutropenia and severe thrombocytopenia) as independent predictors of the risk of death in children and adolescents younger than 15 years of age admitted for VL treatment in a tertiary hospital in northeast Brazil.

Factors related with death or severity of VL were previously assessed in four studies from Africa [10]–[13] and one from Latin America [5]. Only one of them, from Tunisia, exclusively analyzed the pediatric age [10]. A total of 232 children were retrospectively evaluated and seven prognostic factors at hospital admission were identified: visit delayed more than 56 days, fever lasting more than 21 days, normal or low temperature (< 37,5°C according to the authors), hemorrhagic syndrome, hemoglobin rate <5.5 g/dL, sedimentation rate <25 mm and hypoalbuminemia <30 g/L [10]. We found three large studies from the Sudan and Uganda [11]–[13]. In each of them more than 1,000 patients from all age groups were included, but only one described risk factors according to age group [11]. In the subset of Sudanese patients younger than 16 years the following risk factors for death were identified: age <2 years, malnutrition (weight for height <60%), anemia (hemoglobin level <6 g/dL) and splenomegaly (Hackett grade 3–5)[11]. In a study from the Northeast of Brazil (Teresina, Piauí) both children and adults were included in a case-control study. Diarrhea, jaundice, fever for more than 60 days and hematocrit ≤20% were found to be significantly associated with death from VL in a multiple logistic regression model [5].

Out of the six factors identified in the present study, only jaundice and hemorrhagic syndrome were previously reported by other groups [5], [10]. These heterogeneous findings could be explained by the diversity of participants included in the different studies regarding age, access to treatment, setting and *Leishmania* species.

An interesting observation is that a higher risk of death was associated with upper gastrointestinal (UGI) bleeding (OR = 38.3, 95% CI not calculable) on bivariate analysis, but this did not remain significant on multivariate analysis, probably due to the small (5) number of children with UGI bleeding. Thrombocytopenia, but not decreased hemoglobin level was associated with higher risk of death. Although UGI bleeding can be associated with low platelet count or decreased hemoglobin level, respectively as a cause and consequence of bleeding, we were not able to analyze serial blood samples for each patient and only admission samples were included in the model.

The newly generated prognostic scoring system is based on four clinical variables and two laboratorial tests that are usually available in poor resource settings. In addition, it demonstrated satisfactory sensitivity and specificity similarly to those reported in the study held in Teresina, Brazil (sensitivity 85.7% and specificity 92.5%) which also created a prognostic score for children and adults with VL [5].

Our study had limitations that deserve to be discussed. Inherent to retrospective studies, clinical and laboratory data recording might be less accurate, even though our study was carried out in a teaching hospital with regular monitoring of medical recording quality. We have decided to include cases with clinical-epidemiological diagnoses, as well as those with confirmatory tests. While restricting analysis to confirmed cases would be a methodologically interesting option, in real life many cases do not have parasitological confirmation. On the other hand, the case fatality rate (10%) observed in our study was somewhat higher than the national average of 7% [16] and this might be due to the characteristics of this referral hospital that tends to receive cases from the most severe spectrum of disease. The main immediate causes of death were infections and bleeding which are the classical complications of the disease and also compatible with results obtained from other groups in different populations [5], [10]–[12].

Despite these limitations, no previous reports have assessed children-specific independent predictors for death by VL in Latin America. The prognostic score reported in the present study can be promptly assessed in any setting and require only trained health care professionals, therefore are feasible in developing countries where VL is endemic. Furthermore, the laboratory analyses are simple and relatively cheap and can be performed in most health facilities. Isolated risk factors or a combination of them may lead to important clinical decisions such as: platelet or plasma transfusion, use of antibiotics or use of vitamin K. In addition, early identification of unfavorable prognostic factors can facilitate rational allocation of VL cases to be treated as outpatients, admitted to regional hospitals or referred to larger centers with intensive care facilities. Prognostic and severity markers can also be useful for the selection of candidates for clinical trials.

Visceral leishmaniasis is a model for the study of host-pathogen interaction. The biological rationale to explain why some infected individuals will develop any disease instead of asymptomatic seroconversion remains unclear. A similar uncertainty involves understanding the reasons behind a milder or more severe clinical presentation. The predictors of death identified in the present study (jaundice, dyspnea, suspected bacterial co-infection, mucosal bleeding, severe neutropenia and severe thrombocytopenia) can be associated with an aggressive bone marrow involvement, but also seem to be proxy of a systemic, severe, imbalanced response to the pathogen.

Although predictors can help identifying higher risk patients, thus informing practice, studies addressing earlier risk markers would be welcomed. The role of genetic susceptibility and epigenetic interactions modulating host inflammatory response needs further understanding.
